# Associations Between APOE Genotypes and Vascular Pathology Lesions in Alzheimer's Disease: Insights from the NACC Dataset

**DOI:** 10.1002/alz70855_107130

**Published:** 2025-12-24

**Authors:** Rebecca Bernal, Obed Okwoli Apochi, Yannick Joel Wadop Ngouongo, Crystal Wiedner, Biniyam A Ayele, Muralidharan Sargurupremraj, Xueqiu Jian, Habil Zare, Alfred Kongnyu Njamnshi, Nkouonlack Cyrille, José E. Cavazos, Sudha Seshadri, Alfred N. Fonteh, Bernard Fongang, Alice Nono Djotsa, Agustin Ruiz, Margaret E Flanagan, Jayandra Jung Himali, the STAC ‐ African Initiative on Bioinformatics Online Training in Neurodegenerative Diseases (AI‐BOND)

**Affiliations:** ^1^ Glenn Biggs Institute for Alzheimer's & Neurodegenerative Diseases, University of Texas Health San Antonio, San Antonio, TX, USA; ^2^ National Forensic Sciences University, Gandhinagar, Gujarat, India; ^3^ Department of Biochemistry and Structural Biology, San Antonio, TX, USA; ^4^ Glenn Biggs Institute for Neurodegenerative Diseases, University of Texas Health Science Center at San Antonio, San Antonio, TX, USA; ^5^ Glenn Biggs Institute for Alzheimer's & Neurodegenerative Diseases, University of Texas Health Science Center, San Antonio, TX, USA; ^6^ Global Brain Health Institute, San Francisco, CA, USA; ^7^ College of Health Sciences, Addis Ababa University, Addis Ababa, Ethiopia; ^8^ John P. Hussman Institute for Human Genomics, University of Miami Miller School of Medicine, Miami, FL, USA; ^9^ University of Texas Health Science Center at San Antonio, San Antonio, TX, USA; ^10^ The University of Texas Health Science Center at San Antonio, San Antonio, TX, USA; ^11^ Brain Research Africa Initiative, Yaounde, Centre, Cameroon; ^12^ The University of Yaoundé I, Yaoundé, Cameroon, Cameroon; ^13^ Brain Research Africa Initiative, Yaoundé, Cameroon; ^14^ Neurology and Physiology UT Health San Antonio, San Antonio, TX, USA; ^15^ Glenn Biggs Institute for Alzheimer's & Neurodegenerative Diseases, University of Texas Health Science Center, San Antonio, TX, USA; ^16^ Department of Neurology, Boston University School of Medicine, Boston, MA, USA; ^17^ Framingham Heart Study, Framingham, Boston, MA, USA; ^18^ Department of Neurology, University of Texas Health Sciences Center, San Antonio, TX, USA, San Antonio, TX, USA; ^19^ Huntington Medical Research Institutes, Pasadena, CA, USA; ^20^ Glenn Biggs Institute for Alzheimer's & Neurodegenerative Diseases, The University of Texas Health Science Center at San Antonio, San Antonio, TX, USA; ^21^ Department of Population Health Sciences, The University of Texas Health Science Center at San Antonio, San Antonio, TX, USA; ^22^ Department of Biochemistry and Structural Biology, The University of Texas Health Science Center at San Antonio, San Antonio, TX, USA; ^23^ Center for Health Services Research & Development Center for Innovation Quality, Effectiveness and Safety (IQUEST), Michael E. DeBakey VA Medical Center, Housten, TX, USA; ^24^ Section of Health Services Research, Baylor College of Medicine, Houston, TX, USA; ^25^ Joe R. and Teresa Lozano Long School of Medicine, University of Texas Health Science Center, San Antonio, TX, USA; ^26^ Biomedical Research Networking Centre in Neurodegenerative Diseases (CIBERNED), National Institute of Health Carlos III, Madrid, Madrid, Spain; ^27^ Ace Alzheimer Center Barcelona – International University of Catalunya (UIC), Barcelona, Spain; ^28^ Department of Microbiology, Immunology and Molecular Genetics, University of Texas Health Science Center, San Antonio, TX, USA; ^29^ Glenn Biggs Institute for Alzheimer's and Neurodegenerative Diseases, UT Health San Antonio, San Antonio, TX, USA; ^30^ Pathology, San Antonio, TX, USA; ^31^ Graduate School of Biomedical Sciences, University of Texas Health Science Center, San Antonio, TX, USA; ^32^ Department of Population Health Sciences, University of Texas Health Sciences Center, San Antonio, TX, USA; ^33^ Department of Neurology, Boston University Chobanian & Avedisian School of Medicine, Boston, MA, USA; ^34^ Department of Biostatistics, Boston University School of Public Health, Boston, MA, USA; ^35^ The Framingham Heart Study, Framingham, MA, USA

## Abstract

**Background:**

Alzheimer's disease (AD) is known as a progressive neurodegenerative disorder. Evidence suggest that Apolipoprotein E (*APOE*) plays a significant role with AD, but only limited work has examined *APOE* relationships with vascular pathology lesions in the brain. The objective here is to investigate the association between *APOE* genotypes and vascular pathology lesions using the National Alzheimer's Coordinating Center (NACC) dataset and to further explore absent TDP‐43 proteinopathy vs. any TDP‐43 proteinopathy.

**Methods:**

We used NACC data to evaluate the relationship between *APOE* genotype and vascular pathology lesions: arteriolosclerosis, atherosclerosis, large arterial infarcts, lacunes, hippocampal sclerosis of aging, and hemorrhages and microbleeds. Participants were classified by *APOE* genotype into *APOE* ɛ4 carriers (ɛ24, ɛ34, ɛ44), *APOE* ɛ3 homozygotes (ɛ33), and *APOE* ɛ2 carriers (ɛ22, ɛ23). Logistic regression models adjusting for age, sex, self‐reported ancestry, and education were used. We also assessed effect modification by TDP‐43 proteinopathy in the association between *APOE* genotype and each of the vascular lesions. Statistical significance was set at α=0.05.

**Results:**

This study consisted of 7117 participants (mean age 79.1 ± 11.6 years, 47.6% female). Compared to participants with ε3 homozygotes, those carrying at least one *APOE* ε4 allele have higher odds of Arteriolosclerosis (OR [95% CI], p: 1.19[1.09,1.31],<0.001) and hippocampal sclerosis of aging (1.28[1.07,1.53],0.006) while participants with *APOE* ɛ2 carriers had an increased odds of large arterial infarcts (1.71[1.15, 2.55],0.009). We observed significant effect modification by TDP‐43 proteinopathy in the association between *APOE* genotype and arteriolosclerosis (*p* = 0.037) and hippocampal sclerosis (*p* = 0.044). Among those with absent TDP‐43 proteinopathy, *APOE* ɛ4 carriers showed increased odds of arteriolosclerosis (1.37[1.12,1.68],0.002) as compared to ε3 homozygotes, but not among those with any TDP‐43 proteinopathy present, and none of the other associations were statistically significant.

**Conclusions:**

Our findings suggest that carrying at least one *APOE* ɛ4 allele increases the risk of arteriolosclerosis and hippocampal sclerosis, while the presence of at least one *APOE* ɛ2 increased the risk of large arterial infarcts. TDP‐43 proteinopathy modifies these effects, with *APOE* ε4 showing stronger vascular and neurodegenerative associations in those with absent TDP‐43 proteinopathy but not in those with TDP‐43 proteinopathy.

